# Cortical Responses to Mother's Voice in Comparison with Unfamiliar Voice in the First Trimester of Life: A fNIRS Study

**DOI:** 10.1055/s-0044-1788003

**Published:** 2024-08-01

**Authors:** Lurdiana Guimarães Dias, Débora Marques de Miranda, Ana Lívia Libardi Bertachini, Gabriela Cintra Januário, Rebecca Chrispim Silva, Luciana Macedo de Resende

**Affiliations:** 1 Department of Speech-Language Pathology and Audiology, Postgraduate Program in Speech-Language and Hearing Sciences, School of Medicine, Universidade Federal de Minas Gerais (UFMG), Belo Horizonte, MG, Brazil; 2 Department of Pediatrics, Postgraduate Program in Health Sciences of the Child and Adolescent, School of Medicine, Universidade Federal de Minas Gerais (UFMG), Belo Horizonte, MG, Brazil

**Keywords:** spectroscopy, near-infrared, child development, hearing, auditory cortex

## Abstract

**Introduction** The use of functional near-infrared light spectroscopy (fNIRS) may be applied to study cortical responses in children and could offer insight into auditory and speech perception during the early stages of life. Some literature suggests that babies are already able to identify familiar voices at birth, and fNIRS is a non-invasive technique that can be used to study this population.

**Objective** To characterize the cortical responses of infants during their first trimester of life to infant-directed speech using near-infrared light spectroscopy and to verify whether there is a difference in responses when infant-directed speech is performed by their mother compared with an unknown person.

**Methods** Twenty-three children between 0 and 3 months, healthy, without risk indicators for hearing loss, and with results considered normal in the audiological evaluation were tested with near-infrared spectroscopy using infant-directed speech as an auditory stimulus produced by their own mother and by an unknown source.

**Results** Bilateral cortical activation was observed. The responses were present in the temporal, frontal, and parietal regions. Regarding the familiarity aspect, no significant difference was observed for the mother's voice compared with an unknown voice.

**Conclusion** Infant-directed speech has prosodic characteristics capable of activating several cortical regions in the infant's first trimester of life, especially the temporal region. The familiarity effect needs to be better defined for this type of stimulus during this period.

## Introduction

The human auditory system achieves its anatomical structure approximately between the 20th and 23rd week of intrauterine life. 1 2 The system's peripheral and central integrity allows proper sound stimuli access to the auditory cortex, encouraging its maturation. 3

At gestation, the fetus responds to maternal speech by modifying its behavior. 4 Throughout the fetal period, it is constantly exposed to sounds performed by its mother, including her voice and her heartbeat's transmission through the intrauterine environment. After birth, it is expected that the auditory cortex is adapted to maternal sounds; therefore, the mother's voice recognition is facilitated. 5

At birth, infants do not have speech comprehension ability. They do, however, answer the speech signals that are directed toward them. Adults commonly interact with children using a speech register called infant-directed speech (IDS), commonly referred to as “motherese” 6 . This speech signal has unique acoustic properties when compared with a speech typically used among adults, which makes it more attractive to infants 7 . These properties include high fundamental frequency (f0), wider frequency spectrum, slower time than usual, greater rhythm and repetition, as well as a more simplified sentence structure. 6 7 8 The aspects found in IDS do not only provide social and emotional benefits to infants but also promote linguistic processing and assist in language development. 9

The understanding of how auditory and language processing occur at a cortical level within the first years of life with the assistance of objective assessment methods has been increasingly studied over the past years, as demonstrated in a review. 10 As outlined in the cited research, these studies have great potential to offer insights into the cortical responses to auditory stimuli in individuals with standard development, as well as the comparison with those with risks involving hearing and language development, including cases of hearing loss diagnosis. Compared with other exams that assess cortical responses, near-infrared spectroscopy (NIRS) provides a range of advantages. It is a practical exam, with a portable and affordable system, that has a good temporal and spatial resolution, tolerates small head gestures, and does not require sedation or radiation, which makes it suitable for the pediatric population. 3 11 12 13 14 15 16 17

Its functioning is based on the premise that different brain areas are activated throughout tasks performance in which they are involved. Therefore, the neurons' metabolic requirements expand amidst activation, accompanied by oxygen demand and, consequently, cerebral blood flow. 3 13 14 15 17 To supply these demands, there is an increase in oxygenated hemoglobin along with a decrease in deoxygenated hemoglobin. 11

To perform NIRS, sources arranged on the head send light at different wavelengths within the near-infrared spectrum. This light is transmitted through the scalp, given that the skin, bones, and human tissues are relatively transparent to this light range. Since the blood components have various absorption levels, the differences captured by the detectors allow oxyhemoglobin and deoxyhemoglobin concentration measurements, along with the assessment of cortical activity during a task operation. 2 11 15

The present study aimed to use NIRS to describe infants' cortical responses to IDS throughout their first trimester of life and to verify whether there was a significant difference in these responses when IDS was performed by their mother compared with IDS performed by an unknown individual.

## Methods

### Participants

This study consisted of 23 healthy children (based on medical records and legal guardians' statements), without any risk indicators for hearing impairment. Every child underwent auditory assessment, including acoustic immittance measures, Automated Brainstem Auditory Response (ABAR) at 40dBnHL, and Evoked Otoacoustic Emissions (EOAEs) tests, which revealed compatible results within the normal standards for their age.

This study took place at the Molecular Medicine Technology Center and Audiology Research Clinic from the School of Medicine at Universidade Federal de Minas Gerais (UFMG). The present study was approved by the institution's Ethics Committee, no. 3,340,222 (CAAE: 97831218.7.000.5149). The participants' parents were informed about the research, and signed informed consent forms were collected. All procedures followed resolution no. 466/2012 of the Brazilian National Health Council (Conselho Nacional de Saúde – CNS, in Portuguese).

### Stimulus

Two different IDS stimuli were used. For the first stimulus, the child's mother was instructed to spontaneously speak toward the infant, as she would in her daily routine, whereas, for the second stimulus, one of the researchers spontaneously spoke toward the child.

**Fig. 1:**
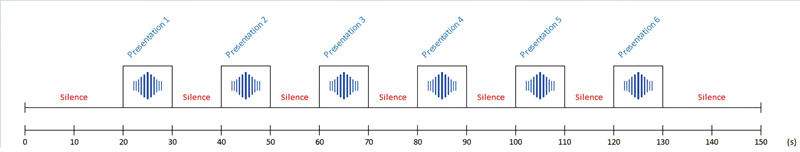
Illustrative demonstration of the auditory stimuli presented for NIRS registration.

Each scenario had a stimulation block with an average of 6 presentations in a minimum period of 10 seconds, followed by a silent period of a minimum of 10 seconds, as well as a 20-second baseline period prior to and after each scenario, as shown in Fig. 1 .

### Data Acquisition

**Fig. 2:**
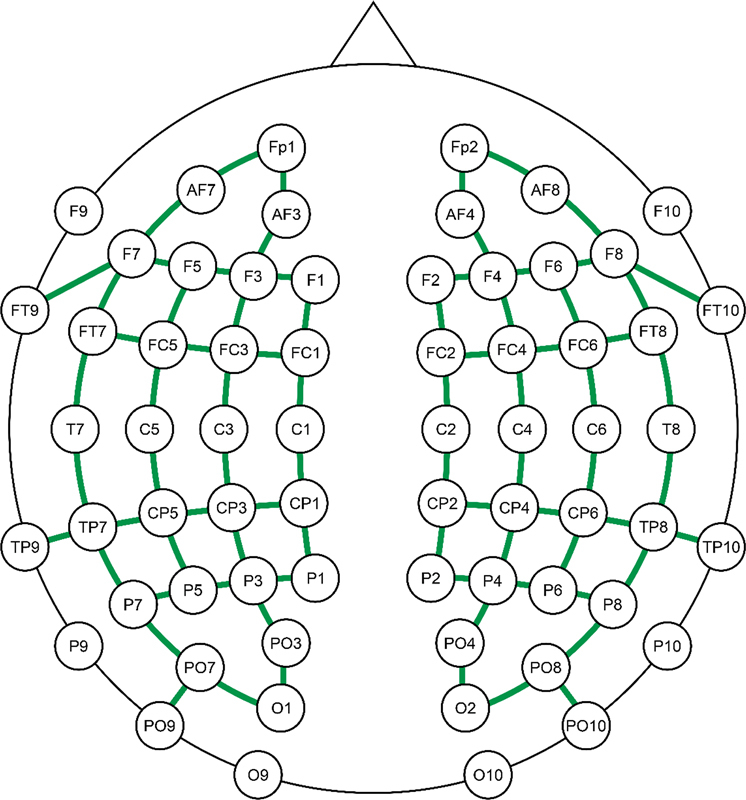
Graphic representation of the optodes/channels distribution in accordance with the 10–20 system.

All measurements were obtained through a continuous-wave NIRS equipment, NIRScout Tandem 1616 (NIRx Medical Technologies, LLC, Glen Head, NY, USA). As a guide for the head cap's position, the child's head circumference measurements were taken, and the Cz scalp location was established with a measuring tape, outlining the nasion-inion and tragus-tragus distances. The international 10–20 system was used as a reference for the optodes placement. An arrangement of 30 light-emitting sources at 2 wavelengths (760 and 850 nm) and 28 detectors were utilized, which established a total of 84 channels positioned on the scalp, covering the frontal, parietal, temporal, and occipital lobes bilaterally ( Fig. 2 ). The distance between each channel's source and detector varied from 1.5 to 2.5 cm, which was determined by their location.

Throughout the experiment, the participants were comfortably lying on their mothers' laps (in a state of rest or being breastfed) and properly positioned to hear the stimuli, ensuring that their ears were not occluded during the stimulation. In the occurrence of any signs of discomfort during the data acquisition, the experiment was interrupted.

The stimuli were presented through a speaker at 60 dBSPL, near the average intensity of human speech. 18 All stimuli followed a standard 40 to 50-cm distance between the device and the child.

### Data Preprocessing

For data preprocessing and analysis, a tool designed for analyzing brain recordings such as NIRS, called the Brainstorm software (open source), was used. 19 20

As a data preprocessing baseline, the designated protocol was previously used in other studies that involved NIRS as the object of study. 21 22 23 The following procedures were performed to assess the hemodynamic response function (HRF) per channel, throughout each block, as well as to exclude those that did not provide sufficient data due to noise or motion artifacts. 21

Primarily, motion artifacts were detected and manually marked. These artifacts are characterized by a signal variation in peak-shaped disturbances that form along the channels. A semi-automatic motion correction was conducted using spline interpolation. 24

The bad functioning channels were automatically detected, which were considered negative values, due to multiple flat segments using a maximum saturation and floor point ratio of 1.00, and the glitches were removed (limit of 2 times the standard deviation).

The oxyhemoglobin (HbO), deoxyhemoglobin (HbR), and total hemoglobin (HbT) concentrations were calculated using the modified Beer-Lambert law. As references for processing options, the following were used: 1 year of age, partial volume effect (PVE) factor of 6, and differential pathlength factor (DPF) method in accordance with Scholkmann and Wolf (2013), 25 based on the mean. Moreover, the linear trend was automatically removed.

A band-pass filter ranging from 0.02 to 0.8 Hz was used to reduce slow systemic physiological hemodynamic instability, high-frequency instrument noise, and rapid oscillations.

The notch filter was applied to eliminate powerline interference at 30 Hz.

MatLab (MathWorks, Natick, MA, USA) codes were used for the detection and automatic exclusion of channels whose intensity changes provided a low signal-to-noise ratio (between the standard deviation and mean greater than 5 within a 5-second time frame) to avoid inadequate recording due to hair thickness. 21

The data were visually inspected, and the channels that still exhibited high amplitude, as well as signal variation with rapid peaks, were removed.

Furthermore, all blocks were visually inspected, and those that still showed motion artifacts were excluded from the analyses. For each stimulus, subjects with more than ⅓ of bad functioning channels (28 channels) or with less than 2 trials for analysis were excluded.

The selected time frame was between -10 thousand and +15 thousand milliseconds from the stimuli onset. Given that the data did not have precisely the same sample range, all of them were resized to 4.0 Hz. For baseline establishment, we used Z-score transformation by selecting the baseline definition from -5 to 0 seconds.

At last, the HRF mean was calculated per infant and per stimulus, for statistical analysis.

### Analysis

The analysis of the results was also performed with the Brainstorm software. Considering that the HRF in the studied age group may present varied patterns (increase or decrease in HbO and HbR concentrations), 26 we chose to apply the power f-test regarding the baseline. Thus, through this test, we sought significant changes in HbO and HbR concentrations in response to auditory stimuli, regardless of negative or positive values. The permutation test was used for the comparison of conditions, making it possible to confirm if the observed differences between groups could have occurred just by chance. Instead of assuming a specific distribution of data, the labels are shuffled off the groups multiple times to see what differences you would expect if there was no true effect. The *p* -value was set at 5%.

## Results

**Fig. 3:**
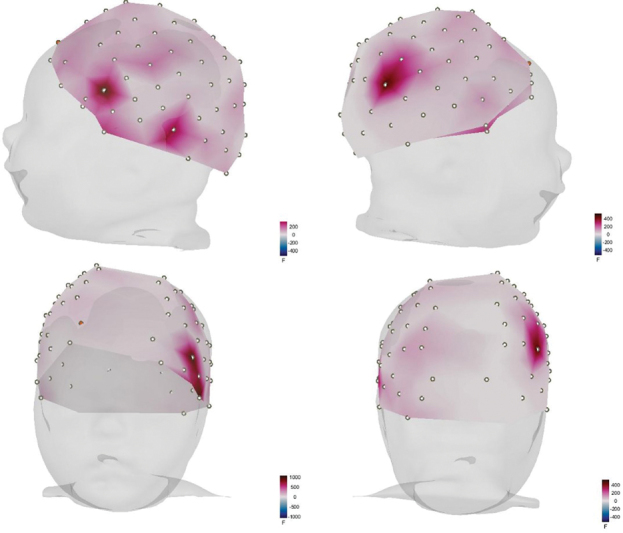
Cortical activation for maternal voice (power f-test).

Fig. 3 demonstrates the cortical activation for the mother's voice given the analysis made by the power f-test. It can be noted that, for this condition, there is a concentrated activation in the posterior area of the right temporal and parietal lobes. However, in the left hemisphere, the area of greatest activation comprises the lower part of the temporal and frontal lobes.

**Fig. 4:**
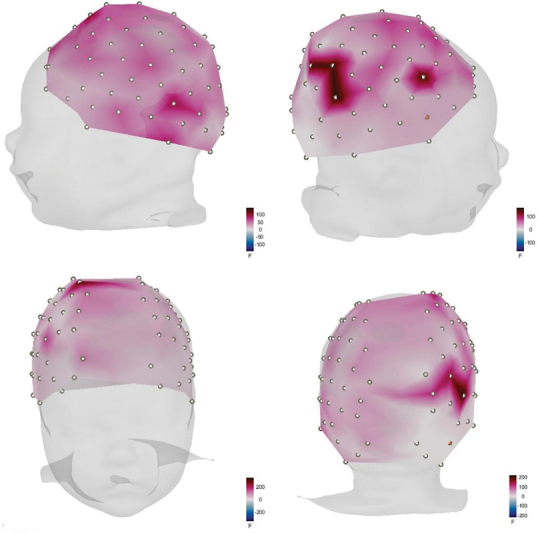
Cortical activation for the researcher's voice (power f-test).

Meanwhile, the power f-test response for the researcher's voice, shown in Fig. 4 , presents a more diffuse activation area, except for the right hemisphere, in which two regions of higher activation are observed within the temporal, parietal, and frontal lobes.

The permutation test, which was conducted to verify if there is a cortical activation difference between the mother's IDS and the IDS of an unknown person, did not show a statistically significant difference.

## Discussion

In the present study, we examined the cortical activation pattern for IDS performed by the child's mother's voice and IDS performed by an unknown voice, as well as the speech familiarity effect in the first trimester of life. The results demonstrated that in both conditions, IDS activates the cortex, particularly the temporal lobe, but also the frontal and parietal areas. Additionally, it should be noted that no statistically significant difference was found when comparing the voice produced by the mother with the voice presented by an unknown person.

As previously mentioned, prosodic patterns demonstrated by IDS are more appealing to this age group. This could be one of the explanations for the bilateral activation of the temporal lobe, as mentioned in another study that also used NIRS as a tool to assess cortical activation for IDS from the mother and from non-acquaintances. 27 In the human brain, the left hemisphere is more prone to present dominance for phonemic processing (vowels and consonants), while the right hemisphere is generally responsible for prosodic processing (rhythm, intonation, emphasis). This specialization facilitates information processing and is known as the lateralization of auditory cortex functions. 28 Therefore, a speech stimulus with rich prosodic characteristics, such as IDS, may be capable of activating both cerebral hemispheres.

As shown in Figs. 3 and 4 , a noticeable cortical activation pattern can be observed within the right hemisphere's temporal and parietal regions, near the supramarginal gyrus area. This cerebral area has been linked in other studies to the detection of sounds with an affective connotation, 29 30 such as IDS. 31 32 This behavior can already be observed in the early days of life, as demonstrated in a study that compared brain activation for speech with emotional prosody and neutral speech in newborns. 30

The brain activity in the frontal area for both conditions is consistent with other studies that found the same pattern. 33 34 In a particular one, this activity in children between 4 and 5 months of age was observed even when the IDS was taken from a voice database with multiple recordings, meaning that the stimulus was not produced by the infant's mother. 34 In another study, similar findings were also found in infants with 2 to 9 days of age. 33 Such results suggest that IDS can be a source of emotional and social stimulation for infants, as the frontal area is directly associated with these parameters. 35

Our study did not find any familiarity difference for IDS, which is consistent with the research conducted by Naoi et al., 27 taking the temporal area into account. However, in the same study, the researchers describe the difference found in the frontal area of the infants' brain, acknowledging that the IDS performed by their mother showed greater activation in this region compared with the IDS of an unknown voice. It should be emphasized that, in that study, the target population had a higher chronological age than the one investigated here (4–13 months of age); therefore, the difference found between them could be explained by a lower specificity of responses in the first trimester of life.

It is known that the human frontal cortex is responsible for functions such as emotions, attention, and affection, 27 35 and the cortical maturation process occurs rapidly during this stage of life, leading to increasingly refined brain areas, to perform their respective functions. 36 Moreover, the fact that these children had more time to experience the relationship with their mothers may explain the discrepancy found between the two studies. However, such an explanation would not be possible considering research that found discrepancies in the temporal and frontal lobes activation while comparing the mother's voice with an unfamiliar voice in newborns. 5

Among the described studies, the one with the largest sample was by Naoi et al. 27 Taking this information into account, it may be necessary for further research to be conducted on a larger population of younger children to clarify the differences between the IDS of the infant's mother and the IDS of an unknown person throughout the first trimester of life.

The limited sample size is likely the main limitation of our work. However, the layout used involved many channels covering the entire cortical area, which allowed us to broaden our understanding of infants' brain activity with the presentation of IDS.

Future research involving a larger sample size and longitudinal follow-up of infants can provide insights into the IDS processing in normal-hearing children and serve as a benchmark for comparison with those with hearing impairment.

Near-infrared spectroscopy has been increasingly used over the past few years. This neuroimaging technique has been proven to be a valuable tool for improving the understanding of the processing and organization of various brain functions in both adults and children. Moreover, it has been particularly useful for the comprehension of human developmental processes. Once again, it was observed that this tool proved to be a reliable option for cortical hemodynamics assessment, especially in infants, in whom other techniques may present limitations that make their implementation challenging.

The next steps to enhance the comprehension of this process entail further research involving a greater cohort of children being longitudinally followed up during their development.

## Conclusion

Among the findings, we observed cortical activation for IDS in the first trimester of life, encompassing multiple areas in both cerebral hemispheres, with prominent activation points in the temporal, parietal, and frontal areas. Regarding the stimulus familiarity for the infant, the results obtained here suggest that there was no significant difference when the source of the stimulus was the mother herself or an unknown person.

## References

[1] Johnson Chacko L Wertjanz D Sergi C Growth and cellular patterning during fetal human inner ear development studied by a correlative imaging approach BMC Dev Biol 2019 19 01 11 10.1186/s12861-019-0191-y 31109306 PMC6528216

[2] Ferreira L De Souza A EH Bertuol B De Melo Â Rechia I C Biaggio E PV Audiometria de reforço visual em lactentes nascidos a termo e pré-termo: Nível mínimo de resposta Distúrb Comun 2016 28 03 492 500

[3] Fontes A A De Miranda D M De Resende L M Espectroscopia de luz próxima ao infravermelho e processamento sensorial auditivo em lactentes Rev CEFAC 2016 18 04 965 973 10.1590/1982-0216201618422615

[4] Marx V Nagy E Fetal behavioural responses to maternal voice and touch PLoS One 2015 10 06 e0129118 10.1371/journal.pone.0129118 26053388 PMC4460088

[5] Uchida-Ota M Arimitsu T Tsuzuki D Maternal speech shapes the cerebral frontotemporal network in neonates: A hemodynamic functional connectivity study Dev Cogn Neurosci 2019 39 100701 10.1016/j.dcn.2019.100701 31513977 PMC6969365

[6] Sulpizio S Doi H Bornstein M H Cui J Esposito G Shinohara K fNIRS reveals enhanced brain activation to female (versus male) infant directed speech (relative to adult directed speech) in Young Human Infants Infant Behav Dev 2018a52 89 96 10.1016/j.infbeh.2018.05.009 29909251 PMC6528784

[7] Spinelli M Fasolo M Mesman J Does prosody make the difference? A meta-analysis on relations between prosodic aspects of infant-directed speech and infant outcomes Dev Rev 2017 44 1 18 10.1016/j.dr.2016.12.001

[8] Fava E Hull R Bortfeld H Dissociating cortical activity during processing of native and non-native audiovisual speech from early to late infancy Brain Sci 2014 4 03 471 487 10.3390/brainsci4030471 25116572 PMC4194034

[9] Kalashnikova M Burnham D Infant-directed speech from seven to nineteen months has similar acoustic properties but different functions J Child Lang 2018 45 05 1035 1053 10.1017/S0305000917000629 29502549

[10] Harrison S C Hartley D E Shedding light on the human auditory cortex: A review of the advances in near infrared spectroscopy (NIRS) Rep Med Imaging 2019 12 31 42 10.2147/RMI.S174633

[11] Anderson C A Lazard D S Hartley D E Plasticity in bilateral superior temporal cortex: Effects of deafness and cochlear implantation on auditory and visual speech processing Hear Res 2017 343 138 149 10.1016/j.heares.2016.07.013 27473501

[12] Anderson C A Wiggins I M Kitterick P T Hartley D EH Pre-operative brain imaging using functional near-infrared spectroscopy helps predict cochlear implant outcome in deaf adults J Assoc Res Otolaryngol 2019 20 05 511 528 10.1007/s10162-019-00729-z 31286300 PMC6797684

[13] Basura G J Hu X S Juan J S Tessier A M Kovelman I Human central auditory plasticity: A review of functional near-infrared spectroscopy (fNIRS) to measure cochlear implant performance and tinnitus perception Laryngoscope Investig Otolaryngol 2018 3 06 463 472 10.1002/lio2.185 PMC6302720 30599031

[14] Bortfeld H Functional near-infrared spectroscopy as a tool for assessing speech and spoken language processing in pediatric and adult cochlear implant users Dev Psychobiol 2019 61 03 430 443 10.1002/dev.21818 30588618 PMC7363196

[15] Bulgarelli C Blasi A Arridge S Dynamic causal modelling on infant fNIRS data: A validation study on a simultaneously recorded fNIRS-fMRI dataset Neuroimage 2018 175 413 424 10.1016/j.neuroimage.2018.04.022 29655936 PMC5971219

[16] McKay C M Shah A Seghouane A K Zhou X Cross W Litovsky R Connectivity in language areas of the brain in cochlear implant users as revealed by fNIRS Adv Exp Med Biol 2016 894 327 335 10.1007/978-3-319-25474-6_34 27080673 PMC5505730

[17] Saliba J Bortfeld H Levitin D J Oghalai J S Functional near-infrared spectroscopy for neuroimaging in cochlear implant recipients Hear Res 2016 338 64 75 10.1016/j.heares.2016.02.005 26883143 PMC4967399

[18] Koishi H U Tsuji D H Imamura R Sennes L U Variação da intensidade vocal: Estudo da vibração das pregas vocais em seres humanos com videoquimografia Rev Bras Otorrinolaringol 2003 69 04 464 470 10.1590/S0034-72992003000400005

[19] Tadel F Baillet S Mosher J C Pantazis D Leahy R M Brainstorm: A User-Friendly Application for MEG/EEG AnalysisComput Intell Neurosci.2011;vol. 2011,ID 879716 10.1155/2011/879716 PMC3090754 21584256

[20] Tadel F Introduction 2021 https://neuroimage.usc.edu/brainstorm/

[21] Ferry A L Fló A Brusini P On the edge of language acquisition: inherent constraints on encoding multisyllabic sequences in the neonate brain Dev Sci 2016 19 03 488 503 10.1111/desc.12323 26190466

[22] Fló A Brusini P Macagno F Nespor M Mehler J Ferry A L Newborns are sensitive to multiple cues for word segmentation in continuous speech Dev Sci 2019 22 04 e12802 10.1111/desc.12802 30681763

[23] May L Byers-Heinlein K Gervain J Werker J F Language and the newborn brain: does prenatal language experience shape the neonate neural response to speech? Front Psychol 2011 2 222 10.3389/fpsyg.2011.00222 21960980 PMC3177294

[24] Scholkmann F Spichtig S Muehlemann T Wolf M How to detect and reduce movement artifacts in near-infrared imaging using moving standard deviation and spline interpolation Physiol Meas 2010 31 05 649 662 10.1088/0967-3334/31/5/004 20308772

[25] Scholkmann F Wolf M General equation for the differential pathlength factor of the frontal human head depending on wavelength and age J Biomed Opt 2013 18 10 105004 10.1117/1.jbo.18.10.105004 24121731

[26] de Roever I Bale G Mitra S Meek J Robertson N J Tachtsidis I Investigation of the pattern of the hemodynamic response as measured by functional near-infrared spectroscopy (fNIRS) studies in newborns, less than a month old: A systematic review Front Hum Neurosci 2018 12 371 10.3389/fnhum.2018.00371 30333736 PMC6176492

[27] Naoi N Minagawa-Kawai Y Kobayashi A Cerebral responses to infant-directed speech and the effect of talker familiarity Neuroimage 2012 59 02 1735 1744 10.1016/j.neuroimage.2011.07.093 21867764

[28] Arimitsu T Uchida-Ota M Yagihashi T Functional hemispheric specialization in processing phonemic and prosodic auditory changes in neonates Front Psychol 2011 2 202 10.3389/fpsyg.2011.00202 21954386 PMC3173826

[29] Köchel A Schöngaßner F Feierl-Gsodam S Schienle A Processing of affective prosody in boys suffering from attention deficit hyperactivity disorder: A near-infrared spectroscopy study Soc Neurosci 2015 10 06 583 591 10.1080/17470919.2015.1017111 25721229

[30] Zhang D Zhou Y Hou X Cui Y Zhou C Discrimination of emotional prosodies in human neonates: A pilot fNIRS study Neurosci Lett 2017 658 62 66 10.1016/j.neulet.2017.08.047 28842278

[31] Adriaans F Swingley D Prosodic exaggeration within infant-directed speech: Consequences for vowel learnability J Acoust Soc Am 2017 141 05 3070 3078 10.1121/1.4982246 28599541 PMC5418129

[32] Parlato-Oliveira E Saint-Georges C Cohen D “Motherese” prosody in fetal-directed speech: An exploratory study using automatic social signal processing Front Psychol 2021 12 646170 10.3389/fpsyg.2021.646170 33790843 PMC8006442

[33] Saito Y Aoyama S Kondo T Frontal cerebral blood flow change associated with infant-directed speech Arch Dis Child Fetal Neonatal Ed 2007 92 02 F113 F116 10.1136/adc.2006.097949 16905571 PMC2675452

[34] Sulpizio S Doi H Bornstein M H Cui J Esposito G Shinohara K fNIRS reveals enhanced brain activation to female (versus male) infant directed speech (relative to adult directed speech) in Young Human Infants Infant Behav Dev 2018b52(June):89 96 10.1016/j.infbeh.2018.05.009 29909251 PMC6528784

[35] Zhang D Zhou Y Yuan J Speech prosodies of different emotional categories activate different brain regions in adult cortex: An fNIRS study Sci Rep 2018 8 01 218 10.1038/s41598-017-18683-2 29317758 PMC5760650

[36] Dean D C III Planalp E M Wooten W Investigation of brain structure in the 1-month infant Brain Struct Funct 2018 223 04 1953 1970 10.1007/s00429-017-1600-2 29305647 PMC5886836

